# Editorial: *Bacillus* spp. - Transmission, pathogenesis, host-pathogen interaction, prevention and treatment

**DOI:** 10.3389/fmicb.2023.1307723

**Published:** 2023-10-19

**Authors:** Piyush Baindara, Bilal Aslam

**Affiliations:** ^1^Department of Radiation Oncology, University of Missouri, Columbia, MO, United States; ^2^Institute of Microbiology, Government College University, Faisalabad, Pakistan

**Keywords:** *Bacillus* spp. - transmission, pathogenesis, host-pathogen interaction, prevention, treatment, *Bacillus*, anthrax, transmission

The *Bacillus* and related genera are some of the most prevalent bacteria in our surroundings and interact with humans via different means, such as soil, air, and plants, and even reside in the human gut. Members of *Bacillus* species are Gram-positive, spore-forming, and facultative aerobes. Interestingly, the *Bacillus* genus contains both pathogens and non-pathogens and has a complex taxonomic relationship. *B. anthracis, B. cereus*, and *B. thuringiensis* are the most studied pathogenic bacteria of the genus *Bacillus* that are known to cause food poisoning, localized infections such as ocular and ear canal infections, and systemic infections such as meningitis and bacteremia. *B. anthracis* is the causative agent of anthrax, an acute infection in humans, economically important livestock, and wild animals. Anthrax is lethal in humans and may cause severe gastrointestinal and pulmonary infections. *B. cereus* is known as one of the food poisoning agents and has been reported to cause localized wound and eye infections in humans. Some *B. thuringiensis* strains are entomopathogens that have been developed as biopesticides and can possibly be transmitted to humans, causing infections in immunocompromised patients.

The pathogenesis of the *Bacillus* species depends upon the survival of spores in the non-host environment, as dormant spores are the main infectious form of the bacterium. Sporulation of *B. anthracis* occurs only in the non-host environment when vegetative cells are exposed to the air and the reasons behind this are not well understood. Furthermore, the environment poses a major challenge for host-to-non-host transition via temperature, moisture, and nutrient availability. Interestingly, in the form of metabolically inactive spores, pathogenic bacteria can endure extreme challenges, including pressure, pH, and ultraviolet rays. Human anthrax epidemiology involves livestock, wildlife, soil, and water. In the soil, *B. anthracis* spores can remain viable for decades and avail a potential source of transmission, causing infection to herbivores while grazing via inhalation or ingestion (Jiranantasak et al., 2022).

On the other hand, *B. cereus* mostly exists in soil and water; however, it has been isolated from various mammals, birds, reptiles, and invertebrates, including the guts of poultry, turtles, and the udders of cattle. *B. cereus* strains have been associated with various food and dairy products, including the staple food rice. Notably, spores are resistant to pasteurization and heat treatment. Furthermore, spore hydrophobicity plays a critical role in surface adherence and later results in recurrent problems in *B. cereus* biofilms in milk tanks (Ehling-Schulz et al., 2019).

Currently, there is no effective treatment available for anthrax; however, antibiotics such as doxycycline and ciprofloxacin are being used as treatments. Other available options are monoclonal antibodies, such as raxibacumab, obiltoxaximab, and BioThrax, an active immunization vaccine. To find novel therapies to treat anthrax, Chiang et al. investigated the effector function of a Toll-like receptor (TLR) agonist, DA-98-WW07, in a mouse model. DA-98-WW07 is a novel TLR2 ligand that has shown promising protection against *B. anthracis* challenge. Additionally, adjunct therapy with a suboptimal concentration of DA-98-WW07 in combination with ciprofloxacin showed improved protection of mice against *B. anthracis* infection.

Granovskiy et al. designed a modified and stable recombinant antigen against anthrax (PA) named rPA83m, which has inactivated proteolytic sites and substituted deamidation sites. In another recent approach to solving the low stability problem with PA, authors used plant virus spherical particles (SPs) as stabilizers of recombinant antigens. Animal studies showed enhanced protection upon the combination of these two compositions: rPA83m and SPs containing recombinant antigens. Stable and high anti-rPA83m antibody titers were reported in guinea pigs against the fully virulent 81/1 *B. anthracis* strain, and rPA83 also maintained its immunogenic properties after storage. Overall, stability, immunogenicity, and protection studies suggested that the combined formulation of rPA83m and SPs could be considered as a potential anthrax vaccine candidate, following clinical trials.

Manoharan et al. isolated a *B. cereus* strain named G9241, which causes anthrax-like diseases. The study found that the G9241 culture supernatant showed hemolytic and cytotoxic activities against several mammalian cells when cultured at 25°C; however, no adverse effects were observed at the physiological temperature of 37°C. Employing various genetic and proteomic tools and techniques, the authors concluded that this unique phenotype of G9241 is a temperature-dependent switch that is regulated by PlcR (a pleiotropic transcriptional regulator) and a quorum-sensing activator PapR, a protease. PapR functions as a limiting factor for the PlcR-driven synthesis of toxin at 37°C, which resulted in non-hemolytic and non-cytotoxic cell-free supernatants. It is notable how *B. cereus* G9241 switches between non-anthrax and anthrax-like phenotypes in a temperature-dependent manner. Furthermore, it is worth investigating the regulation mechanisms of toxin regulators in *B. cereus* and *B. anthracis* that could be a potential therapeutic target to fight against anthrax.

As stated earlier, many *Bacillus* species are known to have beneficial effects for human beings. In a recent study, Xie et al. reported the protective effects of *B. velezensis* ADS024 against *Clostridium difficile* toxin-mediated apoptosis in human colonic epithelial cells. *C. difficile* infection (CDI) produces toxin A and toxin B, which cause inflammation and cell death in the intestine. Patients who had a longer antibiotic treatment exposure showed a high risk of CDI, with symptoms of diarrhea, bloody stool, and abdominal pain. The single-strain live biotherapeutic product (SS-LBP) of ADS024 showed promising results in treating the recurrence of CDI after the completion of standard antibiotic treatment. SS-LBP of ADS024 is demonstrated to prohibit toxin B-mediated apoptosis in human colonic epithelial cells and colonic explants via the inhibition of caspase 3 cleavage. In conclusion, *B. velezensis* ADS024 could be developed as an alternative therapeutic strategy for combatting CDI.

Another study by Zhang et al. isolated the endophytic *Bacillus* strain UTF-33 from the leaves of an acid mold, *Rumex acetosa* L., which was identified as *B. mojavensis* upon characterization. The fermentation filtrate of *B. mojavensis* UTF-33 showed inhibitory effects against the ascomycete fungus (*Magnaporthe oryzae*) that causes rice blast and could be developed as a potential biopesticide.

In conclusion, the *Bacillus* species are closely related to humans and the surrounding environment, which ultimately results in a high impact on human health. Diseases caused by *Bacillus* species are neglected; however, they should be studied in detail. Interestingly, both pathogenic and non-pathogenic *Bacillus* species are in a close complex taxonomic relationship. It is important to address the switch responsible for the transition from non-pathogen to pathogen. Owing to the diverse nature and habitats of *Bacillus* species, it is worth understanding their transmission in detail from host to non-host environment. Furthermore, host-pathogen interaction has not been well studied in the case of *Bacillus* species. Detailed studies could open new avenues of prevention and treatment strategies for pathogenic *Bacillus* species.

## Author contributions

PB: Conceptualization, Investigation, Supervision, Writing—original draft, Writing—review & editing. BA: Supervision, Writing—review & editing.
